# Double-high in palmitic and oleic acids accumulation in a non-model green microalga, *Messastrum gracile* SE-MC4 under nitrate-repletion and -starvation cultivations

**DOI:** 10.1038/s41598-020-79711-2

**Published:** 2021-01-11

**Authors:** Che-Lah Wan Afifudeen, Saw Hong Loh, Ahmad Aziz, Kazutaka Takahashi, Abd Wahid Mohd Effendy, Thye San Cha

**Affiliations:** 1 Faculty of Science and Marine Environment, Universiti Malaysia Terengganu, 21030 Terengganu, Malaysia; 2 Satreps-Cosmos Laboratory, Central Laboratory Complex, Universiti Malaysia Terengganu, 21030 Terengganu, Malaysia; 3 Institute of Marine Biotechnology, Universiti Malaysia Terengganu, 21030 Terengganu, Malaysia; 4 Department of Aquatic Bioscience, Graduate School of Agricultural and Life Sciences, The University of Tokyo, 1-1-1, Yayoi, Bunkyo-ku, Tokyo, 113-8657 Japan

**Keywords:** Biodiesel, Lipids, Fatty acids, Oils

## Abstract

Bioprospecting for biodiesel potential in microalgae primarily involves a few model species of microalgae and rarely on non-model microalgae species. Therefore, the present study determined changes in physiology, oil accumulation, fatty acid composition and biodiesel properties of a non-model microalga *Messastrum gracile* SE-MC4 in response to 12 continuous days of nitrate-starve (NS) and nitrate-replete (NR) conditions respectively. Under NS, the highest oil content (57.9%) was achieved despite reductions in chlorophyll content, biomass productivity and lipid productivity. However, under both NS and NR, palmitic acid and oleic acid remained as dominant fatty acids thus suggesting high potential of *M. gracile* for biodiesel feedstock consideration. Biodiesel properties analysis returned high values of cetane number (CN 61.9–64.4) and degree of unsaturation (DU 45.3–57.4) in both treatments. The current findings show the possibility of a non-model microalga to inherit superior ability over model species in oil accumulation for biodiesel development.

## Introduction

Algal-based biodiesel technology remains an important approach to cope with dependency on a dwindling supply of fossil fuel^1^. Rapid growth, smaller space cultivation, high lipid productivity and suitable lipid profiles have made algal-based feedstock a superior substitute for biodiesel as compared to farming of oleaginous crops like oil seed and oil palm. Several model microalgae species, such as *Chlorella vulgaris*^2^*, Nannochloropsis *sp.^3^,* Chlorella variabilis*^4^ and *Chlamidomonas reinhardtii*^5^ were extensively studied for candidacy in bioreactor plantation for sustainable biodiesel development^6^. On the other hand, there are more than 100 lesser known species of Chlorophyta (green microalgae) and the majority of them are classified as non-model species due to their few studies and limited available literature^7^. Many of these non-model species are believed to possess similar, if not slightly better, oleaginous profiles than model species, which are possibly pointing to alternatives to serve as suitable strains for biodiesel feedstock^8^. One non-model species being studied in the lab was *Messastrum gracile* (previously known as *Ankistrodesmus gracile*) under Selenastraceae, which is a newly classified genera under *Messastrum* gen. nov. and might carry better traits as compared to model species^9^. However, studies on the ability of *M. gracile* for oil accumulation remain limited and unexplored in current literature.

Studies revealed that microalgae are able to produce high amounts of lipid under nitrogen depletion, phosphate limitation, exogenous hormone and high temperature^10–13^. Other than under stress regulation, microalgae are also able to accumulate oil during their stationary and late stationary growth phases (confluent stage). During the lipid accumulation phase, microalgae growth, cell division and photosynthesis rate are inhibited as carbon portioning favors triaclyglyceride (TAG) formation, a type of storage component other than sucrose^14^. Many species, such as *Chlorella vulgaris* and *Chlorella sorokiniana* possess fatty acid profiles of the following lipids: palmitic acid (C16:0), oleic acid (C18:1), stearic acid (C18:0), linoleic acid (C18:2) and α-linolenic acid (C18:3n3), which are suitable base for biodiesel development^15,16^. Conversely, studies showed that alteration of fatty acid profiles would increase the polyunsaturated fatty acid (PUFA) accumulation, which setbacks or further complicates feedstock selection since biodiesel production favors more saturated and monounsaturated fatty acid types for its application^17^.

Nonetheless, changes in fatty acid profiles under certain circumstances are unavoidable. It happens when microalgae try to acclimatize under unfavorable conditions, such as nitrogen starvation^3^. High percentage of lipid production alone is not the sole characteristic for the consideration of biofuel feedstock development. Suitable profiles of lipid types must come together with high lipid production to ensure compatibility for biodiesel application^18^. There are several guidelines and standards that were specifically published, targeting on biodiesel development, such as the European specification EN 14214: 2008, United States specification ASTM D6751, Brazil specification RANP/2008 and Malaysia specification SIRIM MS 123: 2005^1,19^. Of these, the cetane number determination (ignition readiness and combustion performance) and cold filter plugging point (fuel line plugging temperature) have been highlighted as an important evaluation for high quality biodiesel^20^. As such, searching for microalgae with high cetane number fatty acids is one of the encouraging approaches and strategies. However, based on available knowledge, no reports on biodiesel evaluation have been conducted for *M. gracile*.

In the present study, the dynamic production of triacylglyceride (TAG) during nitrate replete (NR) and nitrate starvation (NS) are carefully constructed. *M. gracile* SE-MC4, was cultivated in short (Day 1, Day 2 and Day 3), medium (Day 6 and Day 9) and prolonged (Day 12) exposure to NS conditions, while cell density, chlorophyll, biomass, oil productivity and fatty acid composition were determined throughout the experiments. Furthermore, biodiesel evaluation, such as cetane number (CN), degree of unsaturation (DU) and cold filter plugging point (CFPP) was performed to determine the compatibility of *M. gracile* oil for future biodiesel application.

## Results

### Physiological assessments

**Figure 1:**
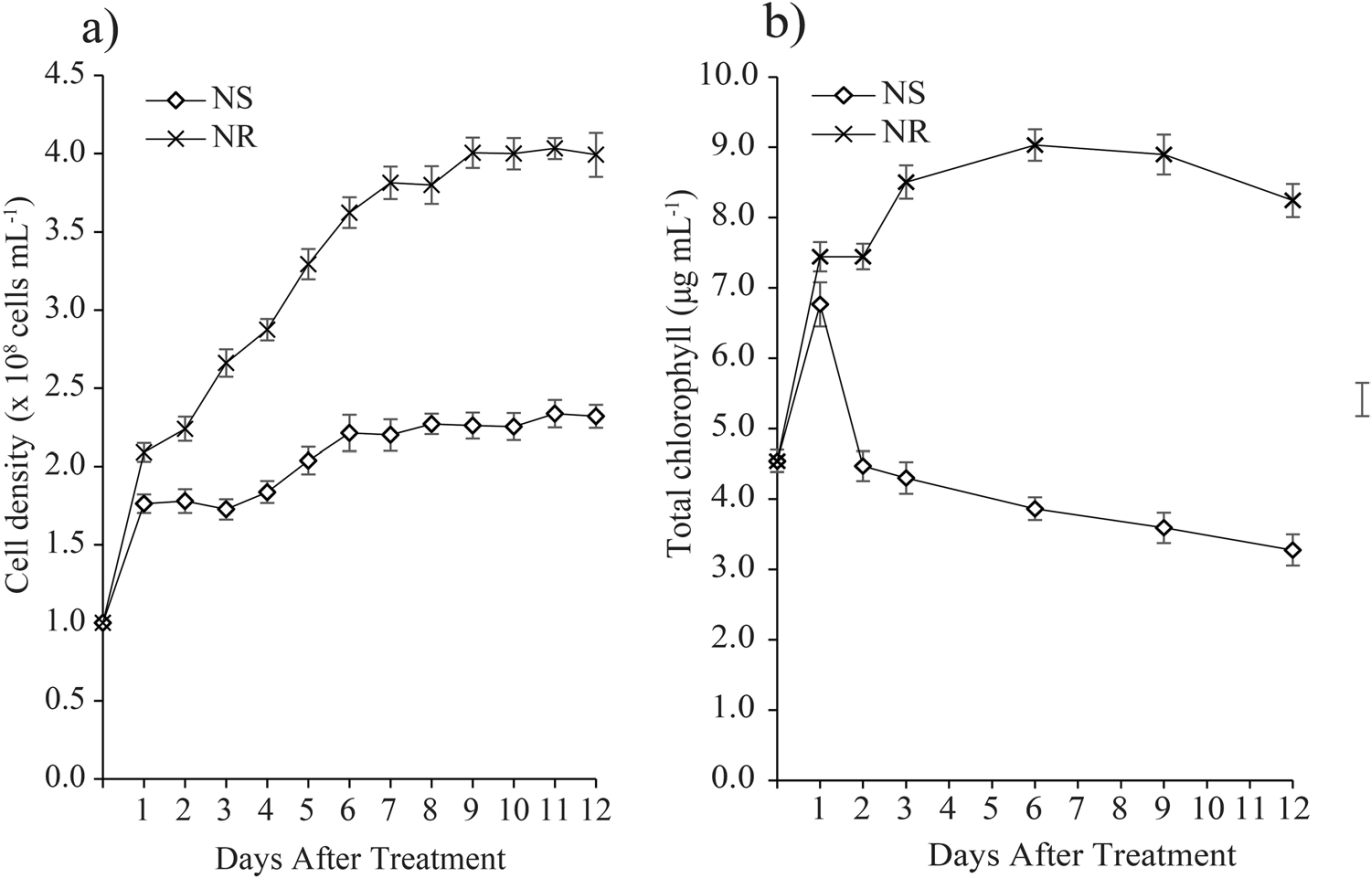
Effects of nitrate starvation on *M. gracile* SE-MC4 cultivation. (**a**) Growth curve of *M. gracile* during 12 days of cultivation under nitrate-starve (NS) and nitrate-replete (NR) conditions. (**b**) Total chlorophyll of *M. gracile* during early (day 1, 2 and 3), mid (day 6 and 9) and prolong (day 12) exposure periods under NS and NR conditions. The values are presented as means ± standard deviation (SD) (n = 3).

Figure 1a depicts the growth performance of *M. gracile* SE-MC4 in NS and NR culture media during Day 12 of cultivation period. Interestingly, a big jump in cell density at Day 1 was observed in both NS and NR cultures, which recorded a 1.8 and 2.1-fold increment, respectively (Fig. 1a). Evidently, the growth performance of *M. gracile* in NR condition tracked the normal exponential growth phase and attained early stationary growth phase on Day 9, whereby no significant cell growth (3.9–4.0 × 10^8^ cells mL^−1^) was observed until Day 12. In contrast, the growth performance of *M. gracile* in NS condition appeared sluggish with a vague and shortened growth phase. The NS cultures entered into early stationary growth phase on Day 6 and rendered a prolonged stationary growth phase of 6 days (Fig. 1a). The growth gap between NS and NR became more evident as the cultures progressed toward a stationary growth phase. A similar steep increase in the total chlorophyll on Day 1 was also observed for *M. gracile* cultured in both NS and NR conditions (Fig. 1b). Thereafter, the trends diverged with NR condition, showing a further increase in chlorophyll content until Day 6 (8.9 µg mL^−1^) before it decreased to 8.2 µg mL^−1^ on Day 12. Conversely, the chlorophyll content in the NS condition continued to decrease after Day 1 to the lowest level of 3.3 µg mL^−1^ on Day 12 (Fig. 1b).

**Figure 2:**
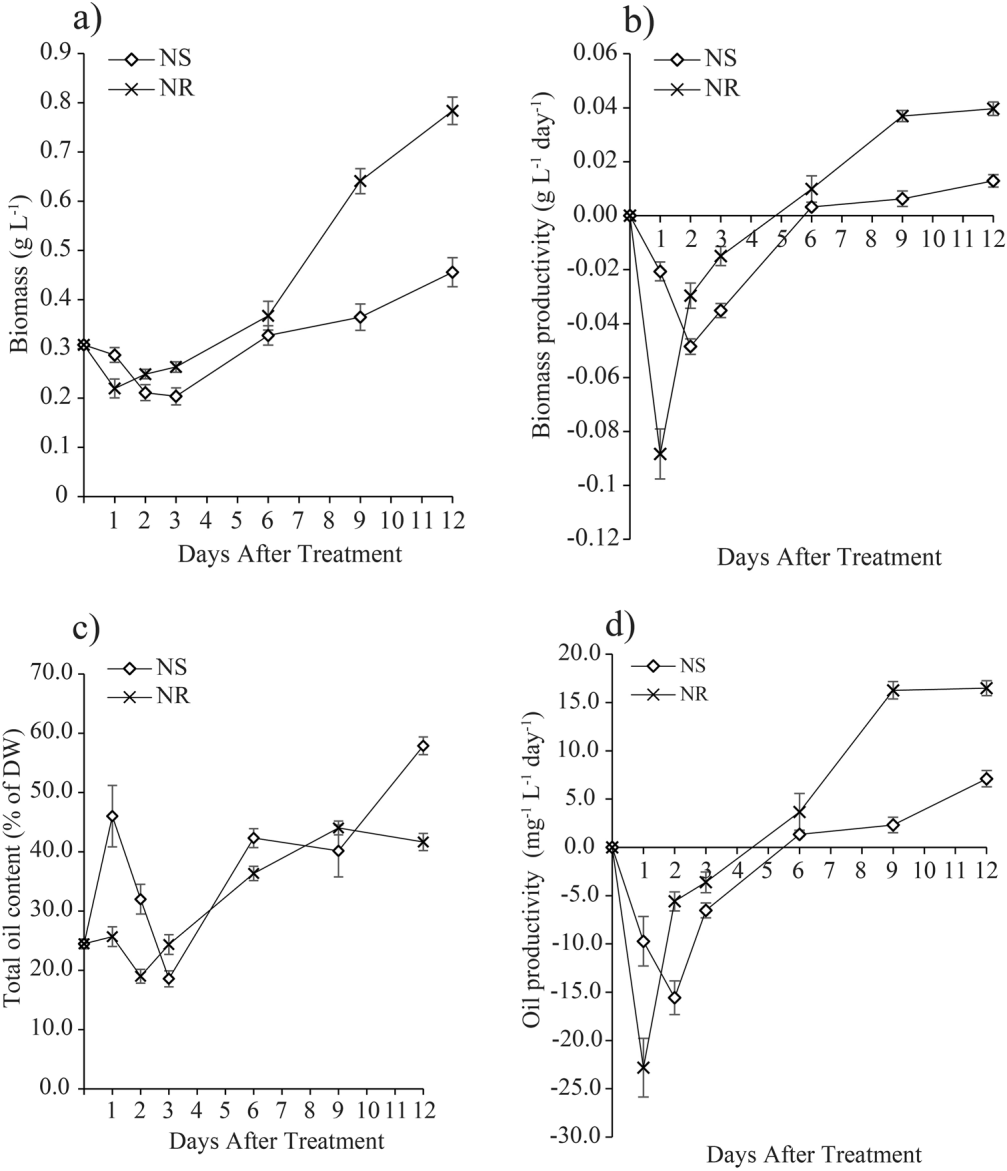
Effects of nitrate availability on *M. gracile* SE-MC4 cultivation during early (day 1, 2 and 3), mid (day 6 and 9) and late (day 12) treatment periods. (**a**) Biomass of *M. gracile* under nitrate-starve (NS) and nitrate-replete (NR) conditions. (**b**) Biomass productivity of *M. gracile* under NS and NR conditions. (**c**) Total oil content of *M. gracile* under NS and NR conditions. (**d**) Oil productivity of *M. gracile* under NS and NR conditions. The values are presented as means ± standard deviation (SD) (n = 3).

The biomass production of *M. gracile* cultured in both NR and NS conditions was lower during the early stage of treatments from Day 1 to Day 3 as compared to the control on Day 0 (Fig. 2a). The declines were well reflected by the negative biomass productivities during the period (Fig. 2b). The biomass productions reversed to an uptrend after Day 2 and Day 3 in the NR and NS cultures, respectively (Fig. 2a) with both achieving a positive biomass productivity on Day 6 and onward (Fig. 2b). As expected, the biomass production in the NR cultures increased drastically from Day 6 to a maximum level of 0.8 g L^−1^ on Day 12, whereas the highest biomass produced by NS cultures was only 0.46 g L^−1^ (or 0.6-fold lower) as compared to the NR cultures on Day 12 (Fig. 2a).

On the contrary, the total oil content of *M. gracile* in NS culture condition was drastically increased from 24.5% on Day 0 to 46.0% (or almost 1.9-fold) on Day 1 of treatment (Fig. 2c). This was followed by a steep decrease into Day 3, whereby it reached its lowest value of 18.5%. However, the inoculation of *M. gracile* cells into new NR media caused a significant drop in the total oil content to 19% on Day 2 as compared to 24.5% on Day 0. The total oil content in the NR and NS cultures bounced back from the low levels on Day 2 and Day 3, respectively. The oil production in both NR and NS cultures continued to trend higher until they achieved the respective highest values of 44.0% (on Day 9) and 57.9% (on Day 12) (Fig. 2c). The oil productivity changes in similar fashion as of biomass productivity throughout the treatment period (Fig. 2b,d), whereby negative oil productivity was recorded during early exposure (Day 1–Day 3) period and a positive oil productivity was achieved on Day 6 and onward in both cultures. The oil productivity continued to trend higher in the NR cultures until it reached a plateau of 16.2 mg L^−1^ day^−1^–16.4 mg L^−1^ day^−1^ on Day 9 and Day 12. However, oil productivity in the NS cultures on Day 9 (2.3 mg L^−1^ day^−1^) and Day 12 (7.1 mg L^−1^ day^−1^) were comparatively lower than the NR cultures (Fig. 2d).

### Fatty acid composition during nitrate-starve and nitrate-replete

There were seven major fatty acids (more than 1% composition) that were detected in both NS and NR conditions, i.e., palmitic acid (C16:0), stearic acid (C18:0), lignoceric acid (C24:0), heneicosylic acid (C21:0), oleic acid (C18:1), α-linolenic acid (C18:3n3) and linoleic acid (C18:2). Several other minor fatty acids also existed, which were negligible and constituted less than 1% of the total oil content, such as caprylic acid (C8:0), lauric acid (C12:0), myristic acid (C14:0), pentadecanoic acid (C15:0), arachidic acid (C20:0) and gondoic acid (C20:1) but were not included in this report.

**Figure 3:**
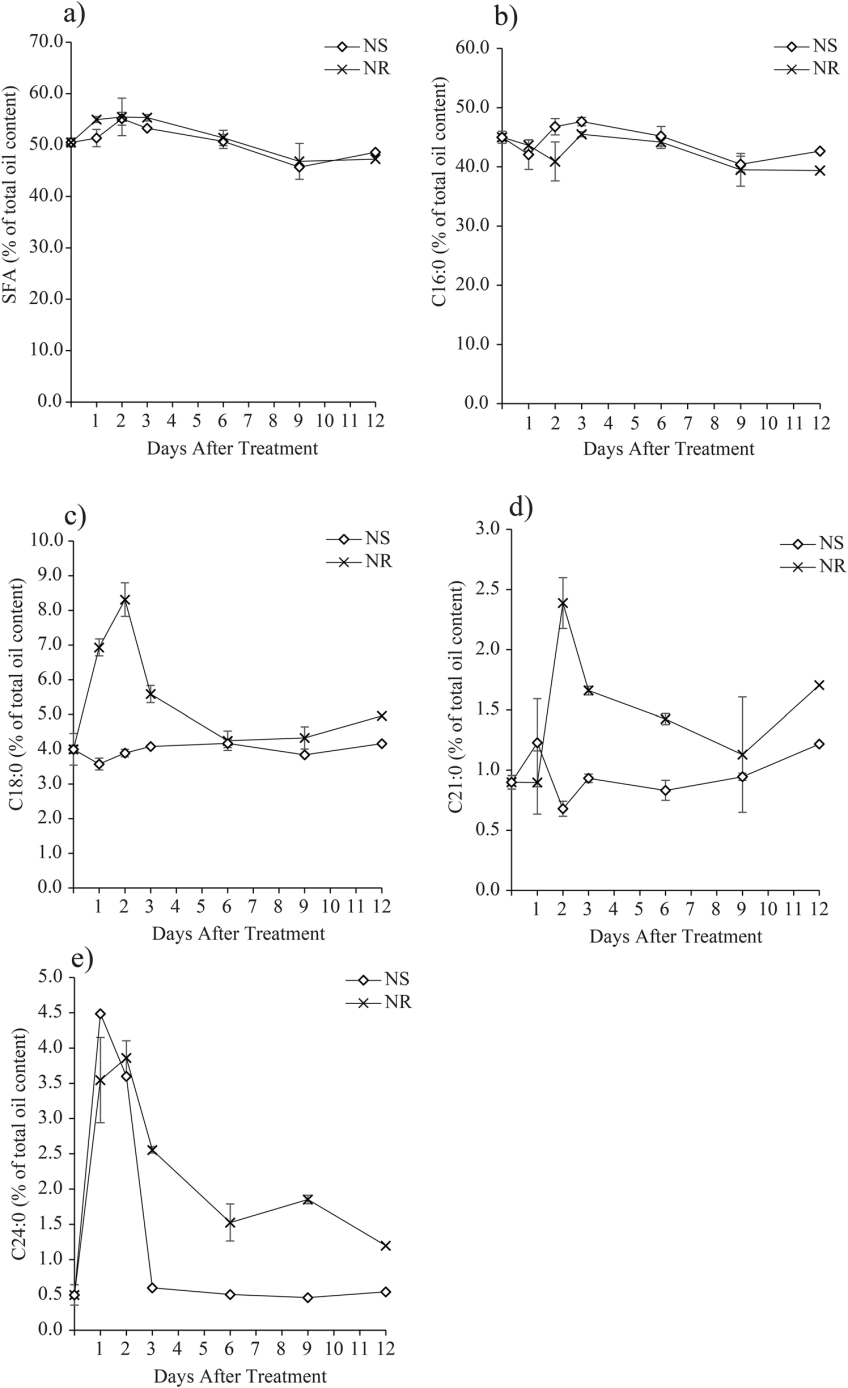
Effects of nitrate availability on saturated fatty acids (SFA) composition of *M. gracile* SE-MC4 cultivation during early (day 1, 2 and 3), mid (day 6 and 9) and late (day 12) treatment periods. (**a**) Overall SFA production, (**b**) percentage of C16:0, (**c**) percentage of C18:0, (**d**) percentage of C21:0, (**e**) percentage of C24:0 of *M. gracile* under NS and NR conditions. The values are presented as means ± standard deviation (SD) (n = 3).

In general, *M. gracile* accumulated relatively higher saturated fatty acid (SFA), which was between 45.7%–55.5% (all percentage of fatty acids were expressed as % of total oil content) as compared to other classes of fatty acids throughout this experiment (Fig. 3a). The SFAs were contributed by C16:0 (Fig. 3b), C18:0 (Fig. 3c), C21:0 (Fig. 3d) and C24:0 (Fig. 3e). It was interesting to note that the accumulation of total SFA followed a similar trend in both NS and NR cultures. Results showed that SFA was significantly increased during early treatment stage (Day 1–Day 3), but with varying pace in both NR and NS cultures. In the NR cultures, the SFA content increased to a maximum level of 55.0% on Day 1 and maintained that level until Day 3, before it gradually decreased to 46.8%–47.3% on Day 9–Day 12. Meanwhile in the NS culture, the SFA content increased to the same maximum level of 55.1% (as of in NR cultures) but only on Day 2 of treatment, before it gradually decreased alongside the NR cultures (Fig. 3a). The C16:0 was the major SFA which accounted between 39.4%–47.6% of total oil content in *M. gracile*. Interestingly, its accumulation pattern differed during the early stage (Day 1–Day 3) of treatments. On Day 2 of the treatment, the C16:0 content declined to 40.9% in the NR culture, against an increment in NS culture to 46.8%. The C16:0 content in both NR and NS cultures was comparable on Day 3 (45.5%–47.6%) and subsequently decreased alongside towards the middle (39.5%–40.5%) and late (39.4%–42.7%) treatment exposures, respectively.

**Figure 4:**
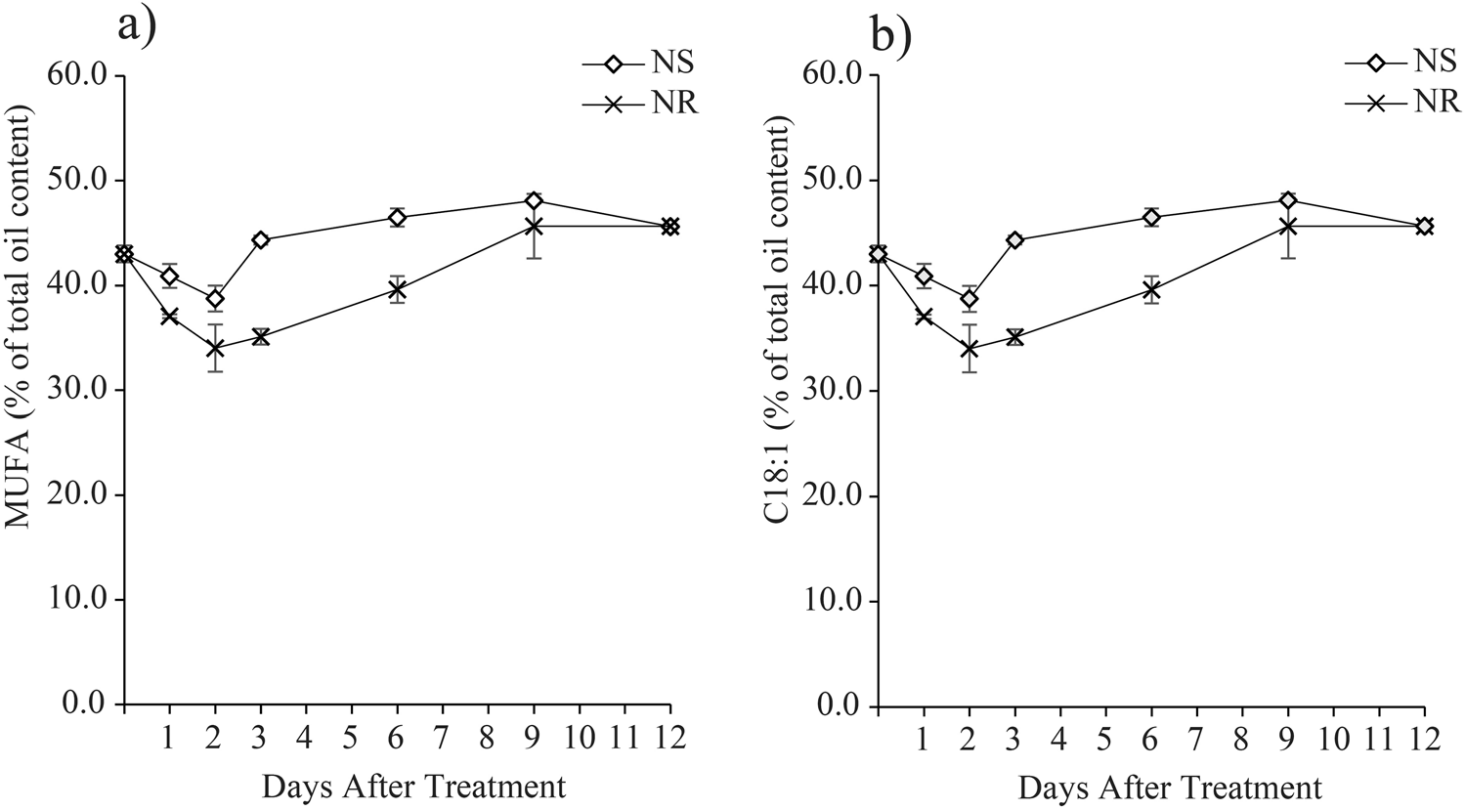
Effects of nitrate availability on monounsaturated (MUFA) composition of *M. gracile* SE-MC4 cultivation during early (day 1, 2 and 3), mid (day 6 and 9) and late (day 12) treatment periods. (**a**) Overall MUFA production, (**b**) percentage of C18:1 of *M. gracile* under NS and NR conditions. The values are presented as means ± standard deviation (SD) (n = 3).

Evidently, the accumulation of minor SFAs, such as C18:0 (Fig. 3c), C21:0 (Fig. 3d) and C24:0 (Fig. 3e) was significantly elevated between 1.7 and 3.6-fold at early stage of treatments (Day 1 and Day 2) in NR as compared to NS cultures. With the exception to C24:0, which was also elevated in the NS cultures on Day 1 and Day 2, nevertheless its content drastically dropped on Day 3–Day 12 to 0.6%–0.5%, a level similar to the Day 0 (Fig. 3e). In the contrary, both C18:0 and C21:0 depicted a minimal fluctuation of 4.0%–5.0% and 0.9%–1.7%, respectively throughout the treatment period. Monounsaturated fatty acid (MUFA) contributed about 34.0% to 48.1% of total lipid composition of *M. gracile* during NS and NR conditions (Fig. 4a). MUFA was contributed by single types of MUFA, which was C18:1 (Fig. 4b). Overall, MUFA in NS cultures was significantly reduced starting from Day 1 to the lowest level of 38.7% on Day 2, while significantly increased on Day 3 onwards to the highest level of 48.1% on Day 9 and slightly reduced to 45.6% on Day 12 of exposure. However, MUFA in NR cultures showed a similar response as in NS at early exposure with a significant decrease during early exposure (34.1%–35.1%), while spiked up starting from Day 6 to the highest level of 45.1% on Day 9 and maintained its level until Day 12 of exposure.

**Figure 5:**
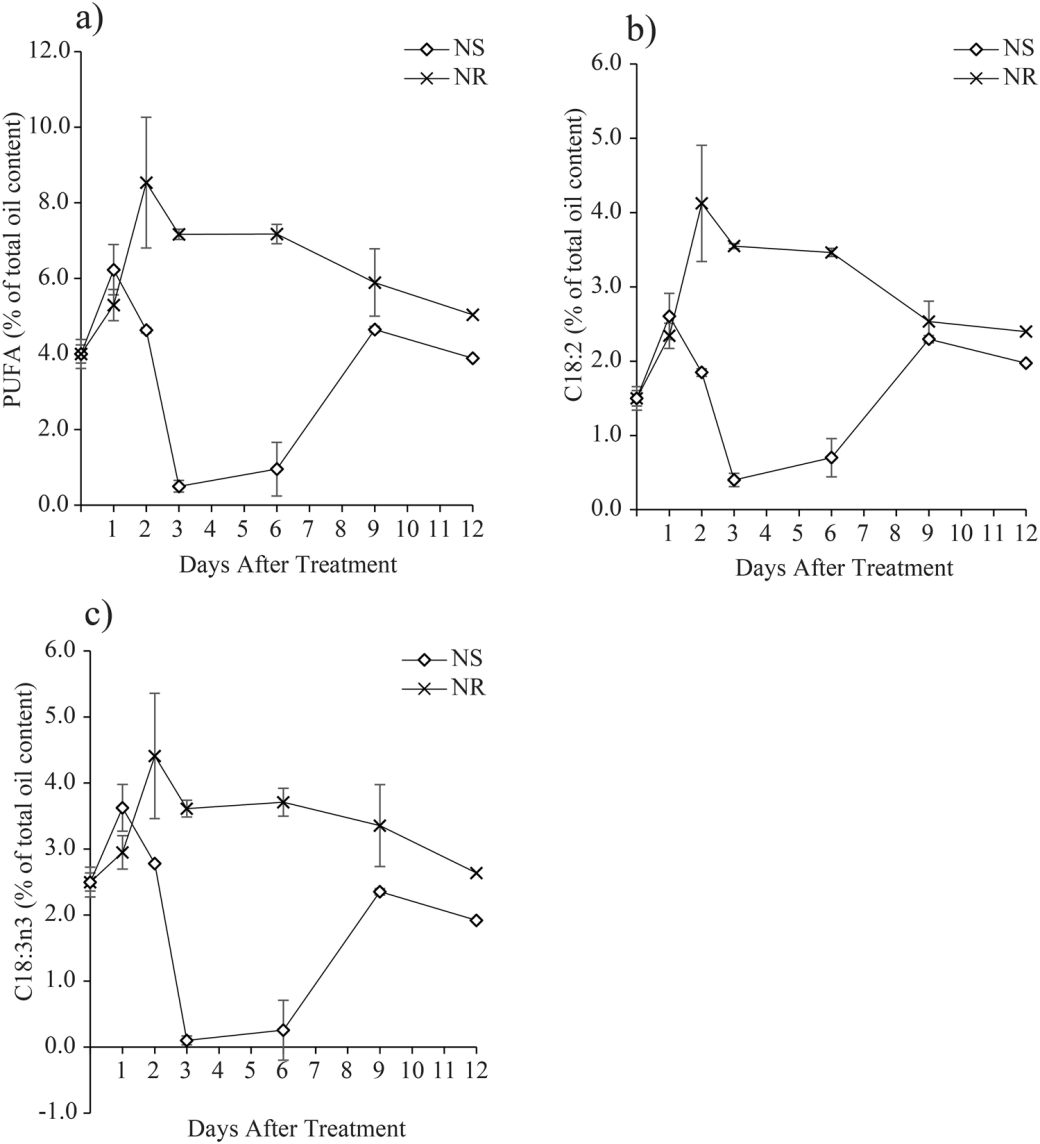
Effect of nitrate availability on polyunsaturated fatty acids (PUFA) composition of *M. gracile* SE-MC4 cultivation during early (day 1, 2 and 3), mid (day 6 and 9) and late (day 12) treatment periods. (**a**) Overall production of PUFA, (**b**) percentage of C18:2, (**c**) percentage of C18:3n3 of *M. gracile* under NS and NR conditions. The values are presented as means ± standard deviation (SD) (n = 3).

*M. gracile* contains relatively lower amount of PUFA, which ranged from 0.5% to 8.5% as compared to other fatty acids, such as SFA and MUFA. The PUFAs were mainly contributed by C18:2 and C18:3n3 under NS and NR cultures. Results revealed that the accumulation of both C18:2 (Fig. 5b) and C18:3n3 (Fig. 5c) followed a similar trend as of the total PUFAs (Fig. 5a) accumulation under NS and NR culture conditions. Interestingly, the trends were diverging starting from Day 2 until they converged back on Day 9 before they declined into Day 12. In the NR cultures, the PUFAs accumulation spiked up on Day 2 to a maximum of 8.5%, followed by a gradual decline into the lowest level of 5.0% on Day 12. In contrast, PUFAs accumulation in the NS cultures spiked on Day 1 to the highest level of 6.2%, while it drastically declined starting from Day 2 to the lowest level of 0.5% on Day 3, before it bounced back to 4.6% on Day 9 and then declined again to 3.9% at late exposure period of NS conditions, which was Day 12.

**Figure 6:**
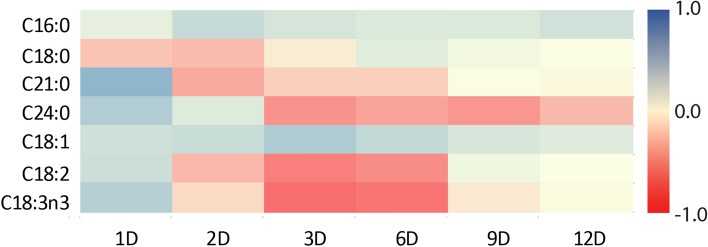
Heat-map plotting of differential fatty acid compositions (fold change ratio) of *M. gracile* SE-MC4 during early (day 1, 2 and 3), mid (day 6 and 9) and late treatment (day 12) of nitrate starve (NS) and nitrate replete (NR) conditions. Positive differential fold changed were in blue color, while negative differential fold changed were in red color scheme.

Heat-map was produced from the log2 fold-change ratio of the fatty acid compositions in the NS cultures normalized to the NR cultures. The blue and red color schemes indicated the highest (1.0) and lowest (− 1.0) fold change, respectively (Fig. 6).The heat-map revealed that all fold-change ratio of fatty acids C16:0, C21:0, C24:0, C18:1, C18:2 and C18:3n3 were higher (blue color) on Day 1 except for C18:0 which declined from Day 1 to Day 3. Interestingly, the declining pattern seen in C18:0 was inherited by C21:0 starting from Day 2 to Day 6 and by C24:0 starting from Day 3 to Day 12 of experiment. However, PUFAs possessed similar trends in log2 fold-change ratio, whereby C18:2 and C18:3n3 possessed lower fold-change ratio starting from Day 2 toward Day 9 (Fig. 5). In contrast, a continuous higher fold-change ratio was observed for both C16:0 and C18:1 fatty acids starting from Day 1 towards the end of the experiment.

### Biodiesel evaluation

In biodiesel evaluation, seven parameters measured were: degree of unsaturation (DU), long chain saturated factor (LCSF), cetane number (CN), saponification value (SV), iodine absorption value (IV) and cold filter plugging point (CFPP). Results revealed that, DU levels followed a similar pattern as of IV values during NS and NR culture conditions. DU and IV were higher on Day 1 of NS culture condition with values of 53.4 gI_2_ 100 g^−1^ and 49.1 gI_2_ 100 g^−1^, respectively (Table 1). However, DU and IV were lower on Day 6 of NS culture condition with values of 48.4 gI_2_ 100 g^−1^ and 41.9 gI_2_ 100 g^−1^, respectively. In contrast, SV and CN possessed independent trends, which differentiated them from each other. SV was significantly higher on Day 1, Day 2, Day 3 and Day 12 (ranging between 199.1 mg KOH g^−1^ and 201.0 mg KOH g^−1^) while CN values were calculated high on Day 2, Day 3 and Day 6 (ranging between 63.3 and 64.4) under NS culture condition. However, CN value in NS was lower as compared to NR culture condition on Day 1 with value of 62.1. On the other hand, LCSF and CFPP managed to show a similar trend, whereby their values were significantly lower in NS compared to NR culture conditions from Day 3 to Day 12 with values ranging between 7.2 °C–8.4 °C and 6.1 °C–9.8 °C, respectively (Table 1).

**Table 1:** Microalgae biodiesel fuel property estimation.

Samples	DU		LCSF		CN		SV (mg KOH g^-1^)		IV (gI_2_ 100 g^-1^)		CFPP ^o^C	
	NR	NS	NR	NS	NR	NS	NR	NS	NR	NS	NR	NS
Day 1	47.6 ± 0.8	53.4 ± 1.0*	15.5 ± 0.5	15.4 ± 0.2	63.2 ± 0.2*	62.1 ± 0.2	198.8 ± 0.1	199.7 ± 0.2*	43.6 ± 0.9	49.1 ± 1.0*	32.2 ± 1.4	32.0 ± 0.6
Day 2	51.1 ± 1.0	48.0 ± 2.3	16.4 ± 1.2	14.3 ± 0.8	62.4 ± 0.2	63.3 ± 0.5*	199.0 ± 0.2	201.0 ± 0.5*	48.0 ± 1.1*	43.8 ± 2.3	35.0 ± 3.9	28.6 ± 2.6
Day 3	49.4 ± 5.6	45.3 ± 1.4	13.0 ± 0.8*	8.4 ± 0.2	62.9 ± 1.0	64.4 ± 0.2*	199.5 ± 0.6	200.7 ± 0.1*	45.8 ± 5.7	39.2 ± 1.2	24.2 ± 2.6*	9.8 ± 0.6
Day 6	54.0 ± 0.5*	48.4 ± 0.1	10.1 ± 0.1*	8.0 ± 0.1	62.5 ± 0.1	63.9 ± 0.1*	200.0 ± 0.2	200.0 ± 0.1	49.7 ± 0.4*	41.9 ± 0.1	15.3 ± 0.4*	8.7 ± 0.1
Day 9	57.4 ± 1.8	57.4 ± 2.1	10.3 ± 0.8*	7.2 ± 0.2	61.9 ± 0.3	62.3 ± 0.4	199.2 ± 0.3	199.0 ± 0.2	52.3 ± 1.7	51.4 ± 2.2	15.9 ± 2.4*	6.1 ± 0.6
Day 12	55.7 ± 4.8	53.4 ± 0.4	9.3 ± 0.4*	7.8 ± 0.1	62.2 ± 0.7	62.9 ± 0.1	198.4 ± 0.1	199.1 ± 0.1*	50.2 ± 4.7	47.7 ± 0.4	12.8 ± 1.1*	8.0 ± 0.2

Estimation includes three fatty acid classes (SFA, MUFA and PUFA), degree of unsaturation (DU), long chain saturated factor assessment (LCSF), cetane number (CN), saponification value (SV), iodine absorption value (IV) and cold filter plugging point temperature (CFPP).

The values are presented as means ± standard deviation (SD) (n = 3). Asterisk (*) represent significant higher using one-way ANOVA (*p* < 0.05) with Fisher’s LSD between NS and NR culture conditions.

## Discussion

### Physiological assessments

Nitrogen is the most abundant nutrient which contributes to a sustainable living ecosystem on Earth. By volume, nitrogen makes up about 78% composition of the air^21^. In the nitrogen cycle, organic nitrogen is passed down to plants in the form of ammonium and nitrate (NO_3_). Autotrophs such as microalgae feed on nitrate (NO_3_^−^) to carry out photosynthesis, biosynthesis of amino acids, DNA replication and biosynthesis of proteins^22,23^. Absence of nitrogen source can disrupt many important cellular functions and enzymes biosynthesis in microalgae, and thus leads to inhibition of growth, cell division and chlorophyll formation. Nitrate is transported into cells via nitrate transporter and further assimilated in chloroplast to produce glutamine. Glutamine is then reduced into glutamate whereby its monomers are used for DNA replication and chlorophyll compartments^24^. Lower cell proliferation and reduction in chlorophylls are unavoidable under nitrate starvation^25^. Furthermore, previous reports showed that glutamates could act as important molecules that facilitate growth, while chlorophyll formation is dependent on intact carbon skeleton of glutamate via C_5_ pathways^26^. Cells need to produce sufficient energy for its pre-organelles, pre-DNA and pre-protein synthesis before being subjected to cell division, while limitation of nitrogen supply will inhibit this preparation. Without the presence of nitrogen or its assimilated form, cells would only depend on available intracellular nitrogen sources. This limited nitrogen source is insufficient to support cell growth and development in the long run (Supplementary Table 1). Unlike conditions of rapid growth under sufficient nitrate concentrations, cell division can still take place with limited nitrate, albeit at a slower rate, while utilizing intracellular nitrate which originates from the gradual degradation of chlorophyll sources^27^. This phenomenon was well observed in *M. gracile* SE-MC4 cultivated under NS conditions. Growth (Fig. 1a) and total chlorophyll (Fig. 1b) were inhibited under NS, and thus proving nitrogen limitation could arrest and disturb cell growth stages and reduce chlorophyll formation. After intracellular nitrogen was deprived, no further cell proliferation could take place which led to growth arrest and the NS culture achieved stationary phase much earlier on Day 6 (Fig. 1a). Furthermore, a drastic drop of 1.7-fold in cell density was also a distinctive sign of nitrogen deprivation in NS culture. Previous research suggested that nitrogen limitation and starvation in *Chlorella* sp. cultures reduced cell density and exposed cells to early experience of stationary phases as compared to sufficient nitrogen supply^10,15^.

While growth is compromised under nitrate limitation, such conditions are conducive toward increasing total oil content, whereby the assimilated carbon will not be utilized for protein biosynthesis, but channeled toward storage in the form of fatty acid or lipid body. In this current study, the biomass and oil productivities of *M. gracile* SE-MC4 were severely affected during early exposure stage in both NS and NR cultures but began to increase rapidly during prolonged exposure stage (Fig. 2b,d). This clearly shows that storage energy (in the form of TAG) catabolism is the earliest response by cells when they enter a new environment. However, levels of TAG will then depend on the availability of substrate and its environment. Stimulation from stress such as lack of substrate will cause the cell to further accumulate more TAG while at the same time sacrificing growth and development processes. Productivity of total oil content is directly proportional to productivity of biomass, and thus giving higher oil production^28^. Microalgae cells accumulate byproducts such as oil as a cognitive mechanism to maintain cellular redox homeostasis. Under stress conditions, higher oxidative stress is unavoidable, and is contributed by non-photochemical quenching (NPQ) in chloroplast^29^. This condition leads to over production of NADPH which possesses high reducing ability. However, neutral lipids such as TAG are also highly reduced molecules and can act as the alternative conformation for storage mechanism. Lipid storage can act as sink for over production of electrons, and thus reducing intracellular oxidative damages and hinders cells from early senescence^10,30^. High lipid storage under stress strategies is measured in other microalgae species, such as in *Chlorella vulgaris*^15^, *Scenedesmus* sp.^31^ and *Scenedesmus obliquus*^32^. However, high lipid storage alone does not capture the overall illustration of the ability of microalgae to be further applied toward biofuel production. Although the total oil content was higher in NS on day 12 as compared to NR (Fig. 2c), the oil productivity in NR was classified as a more efficient approach for oil accumulation in *M. gracile* as it possessed higher oil productivities than that of NS regimes (Fig. 2d). These findings suggested that *M. gracile* does not depend on pretreatment condition as other species of microalgae do, to accumulate high oil content. In other words, a normal cultivation condition is sufficient to achieve both high biomass and high oil productivities in *M. gracile* at its stationary growth phase. In addition, higher biomass was not necessarily coupled with high amount of accumulated oil in *M. gracile* as the oil content remained consistent at days 9 and 12 of the NR cultures (Fig. 2c), against a significant increase in biomass production recorded during the same period of cultivation (Fig. 2a). Previous study has shown that some non-model microalgae species such as *Chlorococcum* sp. TISTR 8583, might possess different strategy in overcoming stress, where it concurrently accumulated both starch and lipid during nitrate limitation^33^.

### Fatty acid composition and biodiesel properties evaluation

Three types of fatty acid classes are: (1) saturated fatty acid (SFA); (2) monounsaturated fatty acid (MUFA); (3) polyunsaturated fatty acid (PUFA). SFA has no double bond, while MUFA possesses a single double bond and PUFA with two or more double bonds^34^. Under normal conditions, *Chlamydomonas reinhardtii* cc-125 possesses 29% SFA, 13% MUFA and 50% PUFA, *Chlorella vulgaris* (40% SFA, 30% MUFA and 30% PUFA) while *Chlorella sorokiniana* has 80% SFA, 19% MUFA and 2.5% PUFA^15,35^. However in the current experiment, *M. gracile* SE-MC4 possessed higher composition of SFA (47–54%) and MUFA (37 – 45%) in the NR culture (Figs. 3, 4). In general, composition of SFA, MUFA and PUFA will vary between species, growth stages and environmental conditions^10^. Reports suggested that, *Chlorella vulgaris* is able to partition more PUFA under low and high nitrate treatment^15^. Even different strains of *Chlamydomonas reinhardtii* produce different composition of fatty acids^35^. This could be due to molecular environments inside the cells which are unique and flexible according to its gene expressions from its genome. Under stress conditions, partitioning of fatty acid composition happens because microalgae cells try to adapt and acclimatize to the stress factor^24,36^. PUFA is seen as a main priority in microalgae lipid synthesis under low and high nitrate treatment due to its role as defense compartment in reducing oxidative stress, and thus reducing effects on organelle membrane stability and cell membrane integrity^37,38^. The main site for lipid partitioning during stress is endoplasmic reticulum, whereby long chain-acyl protein groups (LC-ACP) are transported from chloroplast through membrane contact sites (MCS) or stromules (stroma filled tubules)^39^. LC-ACP will then be partitioned to TAG by Kennedy pathway based on signals transduction, such as Mitogen activated protein kinase (MAPK) signaling cascades during stress environment^40,41^. This adaptation is versatile since microalgae are able to adapt to different environments in a short range of time. Versatility is easily observed in species that occupy the lower clades of taxonomy hierarchy, such as *Scenedesmus obliquus* (considered as intermediate stage in the evolution) which is able to change its normal partitioning from producing high MUFA under Guillard medium to high PUFA in Tamiya medium culture^32^. Interestingly, *M. gracile* SE-MC4 was able to maintain its C16:0 and C18:1 lipids, making the two major fatty acid composition without altering high lipid partition even under NS culture condition (Figs. 3, 4). Lipid partitioning was only observed in PUFA (C18:3n3, C18:2) and long chain SFA (C21:0, C24:0), which made up a total of less than 10% of total fatty acid composition (Figs. 3, 4 and 5). This can be explained by its taxonomy hierarchy, whereby *M. gracile* inhabits the higher clade of the taxonomy tree, and thus expressing lower versatility and more stability in molecular responses are expected^9^. PUFA and SFA were decreased rapidly pointing to reduction in PUFA and long chain SFA (C18:0, C21:0 and C24:0) partitioning of fatty acids in *M. gracile* SE-MC4 under NS condition (Figs. 3 and 6). PUFA and SFA serve as membrane monomer, especially organelles membrane. Previous research suggested that lower partitioning of PUFA and long chain SFA reduced the ability of a cell to perform formation of new organelles, and thus restricting cell proliferation^42,43^, which was evident from the growth curve of *M. gracile* (Fig. 1a).

Quality of biodiesel properties depends on molecular weight of lipid species, number of double bonds and composition of chain length^18^. Good quality oil for biodiesel needs to possess high ignition readiness, good combustion performance and suitable plugging temperature. Several assessments can be used to analyze the FAME output, which are cetane number (CN), degree of unsaturation (DU), long chain saturation factor (LCSF), saponification value (SV), iodine value (IV) and cold filter plugging point (CFPP)^1,44^. CN value is an important assessment measure that determines good oil composition for biodiesel development. CN values can be related to ignition delay, which is the time interval between start of injection and the start of combustion^45^. Higher CN values would improve profiles of heat release, reduce pollutants emission and noise during combustion which are favored for biodiesel development^46^. Several guidelines on biodiesel quality standard (Table 2) for CN value were introduced, such as United States specification (ASTM D6751), European specification (EN 14214), Brazil (RANP/2008) and Malaysia (SIRIM MS 123: 2005)^1,19,20,44^. Formulation of CN determination used in this experiment is the latest update in CN models that possesses more accuracy than previous CN models published in literature^18,44^. Previous reports have shown that microalgae such as *Chlamydomonas sp.* (CN 65), *Scenedesmus obliquus* (CN 64) and *Chlorella vulgaris* (CN 62) are able to produce higher CN value compared to other biodiesel alternatives, such as palm oil (CN 61), peanut oil (CN 53) and soybean (CN 49)^1^.

**Table 2:** Standard biodiesel specification (highlighted only CN and CFPP) between countries and its references.

Country/areas	Specification code	CN	CFPP (°C)	Reference
European	EN 14214: 2008	Min 51	− 18 to 0	^44,47,48^
United States	ASTM D6751	Min 47	− 18 to 3	^44,48^
Brazil	RANP/2008	Min 47	NA	^1^
Malaysia	SIRIM MS 123: 2005	Min 51	Max 15	^19^

The oil produced by *M. gracile* SE-MC4 under both NR and NS conditions were able to generate relatively stable CN numbers ranging between 61 to 65, that devoid from influenced by culture conditions and growth stages (Table 1). Although biomass productivity increase under NR, NS cultures generally produce better CN values than NR. This can be explained by changes caused by production of higher MUFA (Fig. 4) and lower PUFA (Fig. 5) in the fatty acid composition, which is a condition induced under causative nitrogen deficiency. Overall, the FAME produced by *M. gracile* SE-MC4 possesses better CN values in NS conditions which fall within the ranges of all major international biodiesel specification standards (Table 2). In comparison, *M. gracile* possesses higher CN values than other model crops for biodiesel such as peanut (CN 53), olive oil (CN 57), rapeseed (CN 55), soybean (CN 49) and sunflower (CN 50) as stated by Nascimento et al.^1^. Furthermore, the CN values of *M. gracile* are competent with popular microalgae models such as *Chlamydomonas sp.* (CN 65), *Scenedesmus obliquus* (CN 64) and *Chlorella vulgaris* (CN 62)^1^. Higher CN value is directly proportional to lower DU which points to reduction in unsaturated fatty acid composition. Previous studies have revealed that high DU was directly proportional to a longer ignition delay, retarded the start of combustion and increased nitrogen oxide (NOx) emission during combustion which was not suitable for biodiesel development^46,47^. CN value contributes to ignition readiness and good combustion performance while CFPP contributes to suitable fuel like plugging temperature, which is the third character for determination of biodiesel quality properties^1^.

CFPP can be explained as specific temperature at which crystallization of lipid will clog filters and fuel lines. Higher LCSF value is also directly proportional to higher CFPP. Higher CFPP is considered not efficient and not highly recommended for countries with winter seasons as it may contribute to clogging of filter and fuel lines. Previous reports stated that microalgae with higher percentage of high molecular weight SFA will contribute to higher CFPP value, such as *Chlamydomonas sp* (17 °C) and *Scenedesmus obliquus* (11.6 °C)^1^. Due to the high SFA content, the oils produced by *M. gracile* SE-MC4 at early growth phase (Day 2) has high CFPP of up to 35 °C, which proves to be higher than many microalgae species and oil producing crops^1^. However, CFPP was significantly reduced in NS cultures, in particular from day 3–12 where the cells mostly had entered the stationary growth phase. Low CFPP values was due to low LCSF levels that was directly affected by the low C24:0 content in NS compared to NR cultures (Fig. 3e). The fact that during this period, the low CFPP (6.1–9.8 °C) values meet the 15 °C maximum CFPP value requirement for the Malaysia biodiesel standard (SIRIM MS 123:2005), which is significant for its application in hot weather country like Malaysia. High CFPP is unfavorable for biodiesel as lipid crystallization at high temperatures would cause damage to engine systems^1,48^. Nevertheless, fatty acids composition of both NR and NS cultures at stationary growth phase (day 6–12) and in particular at day 12 produce CFPP values that undoubtedly meet the various international specification standards for biodiesel (Table 2). Therefore, *M. gracile* SE-MC4 can be a potential resource for further study and development as biodiesel feedstock for uses in tropical countries, such as Brazil and Malaysia, which have average annual temperatures between 25 and 35 °C, and higher CFPP requirement^1,19^. Additionally, its relatively stable fatty acid composition which is dominated by double-high production of C16:0 and C18:1 under various cultivation conditions as reported in our previous studies^49,50^ and thus current study is an added advantage of this strain. Although the NS conditions produced better quality biodiesel markers, i.e. higher CN value and lower CFPP, its biomass and lipid productivity were compromised. Therefore, further research on increasing the biomass and lipid productivities of *M. gracile* are essentially needed to boost its competency as a superior biodiesel candidate in the near future. Cutting edge technology such as transcriptomic analysis can be employed to harness genes that are responsible for high biomass production during the growth stages.

## Conclusion

A non model microalga, *M. gracile* SE-MC4 was able to accumulate high total lipid content under NS and maintained high oil productivity in NR without changing much of its original fatty acid composition while avoiding the production of unwanted fatty acids in biodiesel application, i.e. DHA and EPA. The data also demonstrated that *M. gracile* SE-MC4 was able to produce competent CN values and CFPP values that gradually improved towards the stationary phase of NS conditions thus opening up a new possibility of *M. gracile* SE-MC4 as potential candidate for biodiesel development and application, preferably suited for temperate countries.

## Methodology

### Cultivation of *M. gracile*

*M. gracile* strain SE-MC4, was previously isolated from Setiu Wetland, Terengganu, Malaysia^51^ and maintained at the microalgae stock collection in Universiti Malaysia Terengganu. Inoculum culture was initiated from a single colony and subsequently cultivated until Day 12 (early stationary growth phase) in 2.5 L of axenic F2 medium in 3.0 L Erlenmeyer flask. Then the cells were harvested and washed with NS medium three times before suspended in a nitrate-starved (NS; 0% of sodium nitrate) F2 medium with high inoculum size of 1 × 10^8^ cells mL^-1^. Cells growth was monitored every day and harvested early (Day 1, Day 2 and Day 3), mid (Day 6 and Day 9) and prolonged (Day 12) periods of nitrate starvation treatment for physiological and biodiesel evaluation. For comparison, the nitrate-replete (NR; 100% of sodium nitrate) F2 medium was used as control. All cultivation processes were incubated under controlled environment at 24 °C ± 2 °C with continuous light-emitting diode (LED) lighting (80 µ mol m^−2^ s^−1^) and aerated with 0.22 µm filtered sterilized air. Three biological replicates (three cultivation flasks) were designated for each time point (Day 1, Day 2, Day 3, Day 6, Day 9, and Day 12). Three replicates were randomly selected in each time point for further physiological assessments.

### Construction of calibration curves for cell density measurement

Cell density calibration curves were constructed using absorbance reading at OD_681,_ which was the optimum absorption wavelength for *M. gracile* using a micro plates reader Varioskan™ LUX (Thermo Fisher Scientific, USA). The number of cells mL^-1^ was obtained using a syringe Liquid Particle Counter-SLS-2000 (Particle Measuring System, USA). A series of a tenfold serial dilution of microalgae cells was prepared for both parameter, which were optical density (OD) and syringe liquid particle counter (SLS). Measurement was taken using three technical and three biological replicates. Linear equation was generated according to constructed a standard curve between values of OD versus number of cells mL^−1^. Equation (1) was further used to determine the cell density of microalgae cultures throughout the experiments.

1 $$ {\text{Cell density }}\left( {{1}0^{{8}} {\text{cells mL}}^{{ - {1}}} } \right) \, = { 4}.{\text{7151x }}{-} \, 0.0{6}0{5} $$

where, x is OD_681_ which was the optimum absorption value for *M. gracile* cells. Cell density value was expressed in 10^8^ cells mL^−1^ unit. OD used was in four decimal places for accuracy.

### Determination of cell growth, chlorophyll content and biomass productivity

The cell growth curve was created by using cell density from day 1 to day 12 of NS and NR cultivation with one day interval. The chlorophyll content and biomass productivity were determined at short (day 1, day 2 and day 3), mid (day 6 and day 9) and prolonged (day 12) exposure time points. For chlorophyll content, one mL of freshly harvested sample was incubated in 100% v/v methanol for 24 h under dark environment at 4 °C. Mixture was then centrifuged at 10,000×*g* at 4 °C for 5 min. Optical density (OD_652.4_ and OD_665.2_) of the supernatant were determined using a micro plates reader Varioskan™ LUX (Thermo Fisher Scientific, USA) and the chlorophyll content was calculated using the Eq. (2)^52^.

$$ {\text{C}}a\left( {\upmu{\text{ g mL}}^{{ - {1}}} } \right) \, = { 16}.{72}\left( {{\text{OD}}_{{{665}.{2}}} } \right) \, {-}{ 9}.{16}\left( {{\text{OD}}_{{{652}.{4}}} } \right) $$

$$ {\text{C}}b\left( {\upmu{\text{ g mL}}^{{ - {1}}} } \right) \, = { 34}.0{9}\left( {{\text{OD}}_{{{652}.{4}}} } \right) \, {-}{ 15}.{28}\left( {{\text{OD}}_{{{665}.{2}}} } \right) $$

2 $$ {\text{Total }}\;{\text{chlorophyll}}\;{\text{ content }}\left( {\upmu{\text{ g mL}}^{{ - {1}}} } \right) \, = {\text{ Chlorophyll}}\; \, a \, + {\text{ Chlorophyll}}\; \, b $$

where, C*a* is chlorophyll a while C*b* is chlorophyll b. C*a*, C*b* and total chlorophyll content were expressed in µg mL^−1^ unit.

The biomass production was determined by harvesting 1.0 L of cell culture via centrifugation and dried at 60 °C until constant weight was achieved. Biomass production was expressed in g L^−1^ unit. The biomass production was further used for volumetric biomass productivity calculation and expressed in g L^−1^ day^−1^. Volumetric biomass productivity was calculated using a mathematical formula (Eq. 3) ^53^.

3 $$ {\text{Biomass }}\;{\text{productivity }}\left( {{\text{g L}}^{{ - {1}}} \;{\text{day}}^{{ - {1}}} } \right) \, = \left( {{\text{B}}_{{2}} {-}{\text{ B}}_{{1}} } \right) \, \div \, \left( {{\text{T}}_{{2}} {-}{\text{ T}}_{{1}} } \right) $$

where, B_2_ and B_1_ are biomass production of final and initial stage respectively. T_2_ and T_1_ are days of final and initial stage respectively. Biomass productivity was expressed in g L^-1^ day^-1^ unit.

### Oil extraction, trans-esterification and FAME analysis

The oil extraction was carried out as previously described by Cha et al.^15^ with slight modification. A total of 0.3 g of dried cells was disrupted with 10 mL concentrated hydrochloric acid in a 50 mL test tube and vortexed for 2 min. The mixture was then boiled in 100 °C water bath for 20 min and subsequently cooled to room temperature. The mixture was extracted once with 12.5 mL of hexane and repeated twice with 7.5 mL of hexane. The hexane extract was collected and placed in a 250 mL flat bottom flask. Hexane solvent was then removed by using a Rotavapor R-300 (Buchi, Switzerland) and the oil content was gravimetrically measured and expressed as dry weight percentage (% DW)^15^. Oil productivity was derived from calculated biomass productivity value with oil content by dry weight percentage value. Determination of oil productivity was carried out using the Eq. 4 as described by Hempel et al.^53^.

4 $$ {\text{Oil}}\;{\text{ productivity }}\;\left( {{\text{mg L}}^{{ - {1}}} \;{\text{day}}^{{ - {1}}} } \right) \, = \, \left( {{\text{BP }} \times {\text{ oil }}\;{\text{content }}\% } \right) \, \times { 1}000 $$

where, BP is the biomass productivity of respected time point. Oil productivity was expressed in mg L^-1^ day^-1^.

Trans-esterification of microalga oil into fatty acid methyl esters (FAME) was carried out using the previously described method^15^. Extracted oil (50 mg) was esterified in a flat bottom Rota vapor flask by adding 4 mL of 0.5 N sodium hydroxide (in methanol) and few boiling chips. The flask was then attached to a Lienberg Condenser and subsequently boiled with heating mantle for 10 min before adding with 5 mL of boron-trifluoride (in 20% v/v methanol). Mixture was heated for 2 min before 2 mL of *n-*heptane was added and then boiled for another 1 min. The flask was removed from the condenser and left to cool in room temperature. After that, 15 mL of saturated sodium chloride was added into the mixture before poured into a test tube. Upper layer was collected into 1.5 mL vial with addition of sodium sulfate anhydrous and ready for gas chromatography fatty acid methyl ester analysis (GC-FAME). Esterified microalga oil was further analyzed using a Shimadzu GC-2010 *plus* gas chromatography with flame-ionization detection (Shimadzu, Japan) fitted with HP-88 capillary column. Chromatography was performed using establish parameters^15^, which using helium as carrier gas with a constant flow rate of 2 mL min^−1^. Injection parameters, split injection was performed with ratio 1:50 at 250 °C respectively. The oven temperatures were set to 175 °C for 10 min and an increased to 250 °C at 3 °C min^−1^ with hold time for 15 min. Annotation of chromatogram peaks was performed by comparing peak and retention time of external reference standard, Supelco 37 Component FAME Mix (Sigma Aldrich, USA).

### Biodiesel fuel property estimation

Biodiesel fuel property of *M. gracile* oil was estimated using FAME as previously described^1,44^. The cetane number (CN) was estimated by using the models (Eq. 5) that involves two independent variables which is chain length and degree of unsaturation. The CN formula was suggested by^44^ as below:

5 $$ {\text{CN }} = {55}.{87 } + \, 0.0{\text{747x}}^{{1}} + \, 0.0{\text{98x}}^{{2}} + \, 0.{\text{164x}}^{{3}} + \, 0.{\text{176x}}^{{4}} {-} \, 0.0{5}0{\text{x}}^{{5}} + \, 0.00{\text{1x}}^{{6}} {-} \, 0.{14}0{\text{x}}^{{7}} {-} \, 0.{\text{273x}}^{{8}} $$

where, x is the percentage composition of FAME in microalga oil (x^1^: C12:0; x^2^: C14:0; x^3^: C16:0; x^4^: C18:0; x^5^: C16:1; x^6^: C18:1; x^7^: C18:2; x^8^: C18:3).

The degree of unsaturation (DU) was determined using models (Eq. 6) that involves percentage composition of saturated fatty acid (SFA), monounsaturated fatty acid (MUFA) and polyunsaturated fatty acid (PUFA) according to^20^.

6 $$ {\text{DU }} = \, \left( {{\text{MUFA}}} \right) \, + \, \left( {{2 } \times {\text{ Cn}}:{2}} \right) \, + \, \left( {{3 } \times {\text{ Cn}}:{3}} \right) $$

where, Cn:2 and Cn:3 represent PUFAs with two and three double bonds, respectively.

Saponification value (SV) (Eq. 7), iodine value (IV) (Eq. 8), long chain saturation factor (LCSF) (Eq. 9) and cold filter plugging point (CFPP) (Eq. 10) were determine by using FAME weight percentage and models generated by^1^.

7 $$ {\text{SV }} = \, \sum \, \left[ {\left( {{56}0 \, \times {\text{ N}}} \right) \, /{\text{ M}}} \right] $$

8 $$ {\text{IV }} = \, \sum \, \left[ {\left( {{254 } \times {\text{ D }} \times {\text{ N}}} \right) \, /{\text{ M}}} \right] $$

9 $$ {\text{LCSF }} = \, \left( {0.{6 } \times {\text{ C16}}} \right) \, + \, \left( {0.{5 } \times {\text{ C18}}} \right) \, + \, \left( {{1 } \times {\text{ C2}}0} \right) \, + \, \left( {{1}.{5 } \times {\text{ C22}}} \right) \, + \, \left( {{2 } \times {\text{ C24}}} \right) $$

10 $$ {\text{CFPP }} = \, \left( {{3}.{1417 } \times {\text{ LCSF}}} \right) \, {-}{ 16}.{477} $$

where, N as percentage composition of FAME, M as molecular weight of respective FAME, D as double bond exist in FAME. The fuel properties (CN, DU, SV, IV, LCSF, CFPP) was generated for each samples from nitrate-starve and nitrate-replete condition.

### Statistical analysis

The effect of NS and NR on physiological changes in *M. gracile* culture was determined in three biological replicates. Comparison between treated (NS) and control (NR) for growth, chlorophyll, biomass production, biomass productivity, oil production, oil productivity and fatty acid composition were plotted as line graph. All data were statistically analyzed by normalization and one-way analysis of variance (ANOVA), while significant differences was determined using post-hoc test (Tukey’s HSD) test with *p* < 0.05. For heat-map analysis, fatty acid compositions from both NS and NR were log2-transformed and divided (NS/NR) and minus by 1 to produce log2 fold change ratio. Units from log2 fold change ratio was converted to color scheme units and values was disregard from stated in the heat-map using Microsoft Excel 2017. Statistical analysis was conducted using Statistical Package for Social Sciences (SPSS, IBM) while Microsoft Excel (Microsoft Office 2017) was used for heat-map analysis.

## Supplementary Information

Supplementary Table S1.

## Data Availability

The datasets generated during and/or analyzed during the current study are available from the corresponding author on reasonable request.
