# Supplementary material for: Double-high in palmitic and oleic acids accumulation in a non-model green microalga, Messastrum gracile SE-MC4 under nitrate-repletion and -starvation cultivations
Source: Sci Rep. 2021 Jan 11;11:381. doi: 10.1038/s41598-020-79711-2 (PMC7801397; doi:10.1038/s41598-020-79711-2)
Supplement: Supplementary file 1 — Supplementary Table S1. [file 41598_2020_79711_MOESM1_ESM.docx]

**Supplementary Materials**

**Double-high in palmitic and oleic acids accumulation in a non-model green microalga, *Messastrum gracile* SE-MC4 under nitrate-repletion and –starvation cultivations**

**Author names and affiliations:**

Che-Lah Wan Afifudeen^2, 3^, Saw Hong Loh^1^, Ahmad Aziz^1^, Kazutaka Takahashi^4^, Mohd Effendy Abd Wahid^2, 3^, Thye San Cha^1, 2, 3*^

^1^Faculty of Science and Marine Environment, Universiti Malaysia Terengganu, 21030 Terengganu, Malaysia

^2^Satreps-Cosmos Laboratory, Central Laboratory Complex, Universiti Malaysia Terengganu, 21030 Terengganu, Malaysia

^3^Institute of Marine Biotechnology, Universiti Malaysia Terengganu, 21030 Terengganu, Malaysia

^4^Department of Aquatic Bioscience, Graduate School of Agricultural and Life Sciences,
The University of Tokyo, 1-1-1, Yayoi, Bunkyo-ku, Tokyo, 113-8657, Japan

** Corresponding author: Cha TS.*

Tel: +609-6683394

Fax: +609-6683193

E-mail: cha_ts@umt.edu.my

**Supplementary Table 1.** Sodium nitrate concentration in medium during NS and NR cultivation.

| Sodium nitrate in culture medium (mM) | | |
| --- | --- | --- |
| Days of cultivation | NS | NR |
| 0 | 0.00 | 0.88 |
| 1 | 0.00 | 0.69 |
| 2 | 0.00 | 0.52 |
| 3 | 0.00 | 0.35 |
| 6 | 0.00 | 0.11 |
| 9 | 0.00 | 0.01 |
| 12 | 0.00 | 0.01 |
